# Aortic Single-Cell Transcriptome Analysis Reveals ApoE-Isoform-Specific Influences on Vascular Disease

**DOI:** 10.3390/ijms27125619

**Published:** 2026-06-22

**Authors:** David Y. Hui, Jeyashree Alagarsamy, April Haller, Mario Medvedovic, Anja Jaeschke

**Affiliations:** 1 Department of Pathology and Laboratory Medicine, University of Cincinnati College of Medicine, Cincinnati, OH 45237, USA; alagarje@ucmail.uc.edu (J.A.); cannonam@ucmail.uc.edu (A.H.); anjajaeschke227@gmail.com (A.J.); 2 Department of Biostatistics, Health Informatics, and Data Sciences, University of Cincinnati College of Medicine, Cincinnati, OH 45267, USA; medvedm@ucmail.uc.edu

**Keywords:** apolipoprotein E, atherosclerosis, single-cell transcriptome, intracellular lipid accumulation, inflammation

## Abstract

The human *APOE* gene is polymorphic with three major alleles that encode apolipoprotein (apo) E2, apoE3, and apoE4. Both apoE2 and apoE4 are associated with increased atherosclerosis risk. This study utilized human *APOE2*, *APOE3*, and *APOE4* gene replacement mice and single-cell RNA sequencing approach to delineate the mechanisms underlying this association. The human *APOE2*, *APOE3*, and *APOE4* mice were fed a Western-type high fat–cholesterol diet for 16 weeks. Hyperlipidemia and significant atherosclerosis were observed in *APOE2* mice but not in *APOE3* or *APOE4* mice. However, increased vascular inflammation was observed in both *APOE2* and *APOE4* mice. Single-cell RNA sequencing followed by cluster analysis identified 25 major cell types in the aorta that include various immune cell types, endothelial cells, and various vascular mural cell subsets. Results showed that cells from the *APOE2* mice were enriched with genes associated with intracellular lipid accumulation and inflammation, whereas cells from the *APOE4* mice displayed elevated oxidative- and/or endoplasmic reticulum-stress and inflammatory response. Thus, apoE2 accelerates atherosclerosis by inducing diet-induced hyperlipidemia and inflammation, while apoE4 does not induce hyperlipidemia but enhances inflammation that may prime the vasculature for atherosclerosis development. The distinct mechanisms by which apoE2 and apoE4 promote atherosclerosis underscore the importance of including apoE genotype information in the design of therapeutics for atherosclerosis intervention.

## 1. Introduction

The human *APOE* gene is polymorphic with three major allelic variants, ε2, ε3, and ε4, encoding apolipoprotein (apo) E2, apoE3, and apoE4, respectively. These apoE isoforms differ from each other by single amino acid substitutions, with the most common isoform apoE3 containing cysteine at residue 112 and arginine at residue 158 of the 299-residue protein. The apoE2 isoform contains cysteine residues at both position 112 and 158 whereas the apoE4 isoform contains arginine residues at both positions. These amino acid substitutions in the primary sequence lead to differences in structural conformation that alters its functional properties. In particular, the R158C substitution in apoE2 results in a protein that is defective in binding to the LDL receptor. In contrast, the C112R substitution in apoE4 alters interaction between the 22-kDa N-terminal domain and the 10-kDa C-terminal domain of the protein, leading to a misfolded protein conformation that causes oxidative stress [1]. The dysfunctional apoE2 increases the risk and severity of coronary artery disease in diabetes as well as the risk of peripheral vascular disease in non-diabetes [2,3,4,5,6]. The dysfunction in apoE4 also increases the overall risk of cardiovascular disease [7,8]. Interestingly, while the ε4 allele is associated with significant increase in risk of Alzheimer’s disease [9], the ε2 allele is associated with lower Alzheimer’s disease risk compared to subjects with the ε3/3 genotype [10,11,12,13].

The association of apoE polymorphism with a plethora of disease risks is due to its expression in a wide spectrum of tissues and cell types. In the central nervous system, apoE is expressed and secreted by glia cells and astrocytes to activate signaling pathways in neurons in an isoform-dependent manner [14,15]. The high potency of apoE4 to stimulate neuronal signaling and amyloid precursor protein synthesis, whereas apoE2 is the least potent isoform, may be one mechanism underlying the differential Alzheimer’s disease risk associated with these apoE variants [15]. Additionally, single-cell transcriptome profiling studies revealed that apoE variants expressed endogenously in cells within the central nervous system also have direct autonomous effects in cell activation and functions that influence Alzheimer’s disease pathology. In particular, apoE3 expressed in microglia and central nervous system-associated macrophages promotes antigen presentation and interferon pathways to reduce amyloid pathology and associated toxicity, whereas apoE4 expression in these cell types downregulates complement and lysosomal pathways to promote stress-related responses [16].

In addition to cells in the central nervous system, apoE is also expressed in hepatocytes, adipocytes, myeloid-lineage immune cells, hormone-producing cells, and vascular smooth muscle cells. ApoE expressed in these cell types also has direct cell autonomous influence as well as indirect influence on cardiometabolic disease risk (reviewed in [17]). ApoE synthesized in the liver is secreted into the plasma circulation in association with lipoproteins where it mediates lipid transport to other tissues and clearance by the liver. Defective apoE expression exemplified in *ApoE^-/-^* mice results in overt hyperlipidemia that leads to robust atherosclerosis [18]. The R158C mutation in apoE2 reduces LDL receptor binding avidity and often leads to dyslipoproteinemia, which increases vascular disease risk [19]. Interestingly, although the C112R mutation in apoE4 has minimal effect on its receptor binding activity, apoE4 binding to cognate apoE receptors does not activate endothelial nitric oxide synthase and impedes endothelial repair after injury to promote vascular disease [20]. In macrophages, apoE synthesized endogenously has autonomous effects on cell functions, including the promotion of cholesterol efflux and limitation of inflammatory responses [21,22]. The importance of macrophage-derived apoE in atherosclerosis protection was demonstrated in bone marrow transplantation experiments in which reconstitution of macrophage apoE expression prevented atherosclerosis in *ApoE^-/-^* mice, and transplantation of bone marrow from *ApoE^-/-^* mice exacerbated atherosclerosis in wild-type mice [23,24]. The role of macrophage apoE in atherosclerosis protection is also isoform-dependent. Whereas apoE3 expressed in macrophages is protective against atherosclerosis, macrophages expressing either apoE2 or apoE4 are not atheroprotective [25]. The inability of macrophage apoE2 and apoE4 to protect against atherosclerosis is due to the autonomous pro-inflammatory properties of these apoE variants [25]. Interestingly, apoE2 and apoE4 promote macrophage inflammation via different mechanisms: whereas apoE2 promotes inflammation due to reduced cholesterol efflux from macrophages, apoE4 exacerbates macrophage inflammation by increasing oxidative stress [25,26].

ApoE is also expressed in smooth muscle cells in the vessel wall. Earlier studies showed that apoE is expressed primarily in quiescent contractile smooth muscle cells but minimally in proliferating smooth muscle cells with a synthetic phenotype [27]. Additional studies revealed that apoE directly influences smooth muscle cell proliferation [28,29], thus suggesting that apoE expression in vascular smooth muscle cells may also influence atherogenesis. However, whether apoE variants have differential autonomous influence on vascular smooth muscle cells in vascular disease protection remains unknown. In the current study, we first compared human *APOE* gene replacement mice, expressing either human *APOE2*, *APOE3*, or *APOE4* in place of the mouse *ApoE* gene at the same locus, for their susceptibility to Western diet-induced atherosclerosis. Aortic cells were also prepared from Western diet-fed human *APOE2*, *APOE3*, and *APOE4* gene replacement mice for single-cell transcriptome profiling to ascertain whether apoE variants differentially modulate immune cell infiltration and smooth muscle cell phenotype subsets in influencing atherosclerosis.

## 2. Results

### 2.1. Human apoE Polymorphism Differentially Impacts Western Diet-Induced Atherosclerosis in Mice

**Figure 1:**
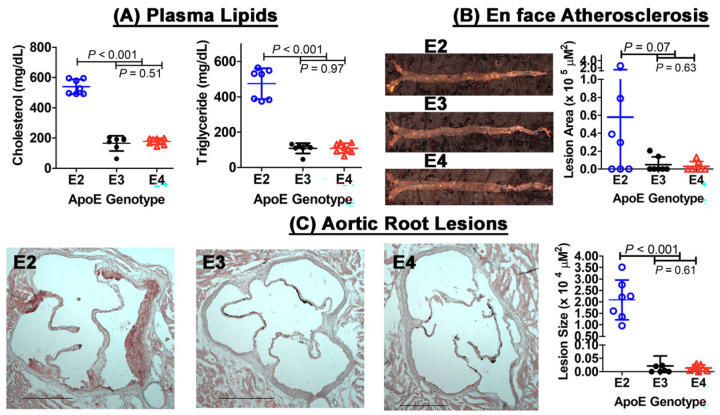
ApoE isoform-specific modulation of plasma lipids and atherosclerosis. Human *APOE2*, *APOE3*, and *APOE4* gene replacement mice (*n* = 7 per group) were fed Western diet for 26 weeks. (**A**) Plasma cholesterol and triglyceride levels were measured from fasted mice. (**B**) Representative images and quantification of Oil Red O-stained atherosclerotic lesions in the whole aorta. (**C**) Representative images and quantification of lesion sizes in the aortic roots. The scale bars represent 500 µm. All plasma lipids and morphometric data passed the Shapiro–Wilk test for normality and were analyzed by one-way ANOVA with Student–Newman–Keuls post hoc analysis with *p* values as shown.

Previous studies have reported that, in comparison to *APOE3* mice, *APOE2* mice displayed increased atherosclerosis and xanthomas while *APOE4* mice also showed a trend toward more atherosclerosis when these animals were fed an atherogenic diet containing very high cholesterol content and sodium cholate [30,31]. In the current study, the *APOE2*, *APOE3*, and *APOE4* mice were fed a Western-type high-fat diet with 0.2% cholesterol that mimics the typical diet consumed by the human population in industrialized society. Although this diet caused only moderate elevation of plasma cholesterol level without noticeable atherosclerotic lesions in the vessel wall of C57BL/6J wild-type mice, plasma cholesterol and triglyceride levels were significantly higher in *APOE2* mice compared to *APOE3* and *APOE4* mice after 12 weeks (Figure 1A). When the animals were sacrificed after 16 weeks on a Western diet, *en face* analysis of the whole aorta revealed a trend toward increased atherosclerosis lesion area in *APOE2* mice compared to *APOE3* and *APOE4* mice, but the difference did not reach statistical significance (Figure 1B). However, analysis of cross-sections of the aortic roots revealed significant increase in lesion size indicative of thicker lesions in *APOE2* mice compared to *APOE3* and *APOE4* mice (Figure 1C). These observations suggest that the human *APOE* gene replacement mice are suitable models to explore the impact of apoE polymorphism on the early phase of Western diet-induced atherogenesis.

### 2.2. ApoE Isoform-Specific Differences in Vascular Gene Expression in Response to Western Diet Feeding

**Figure 2:**
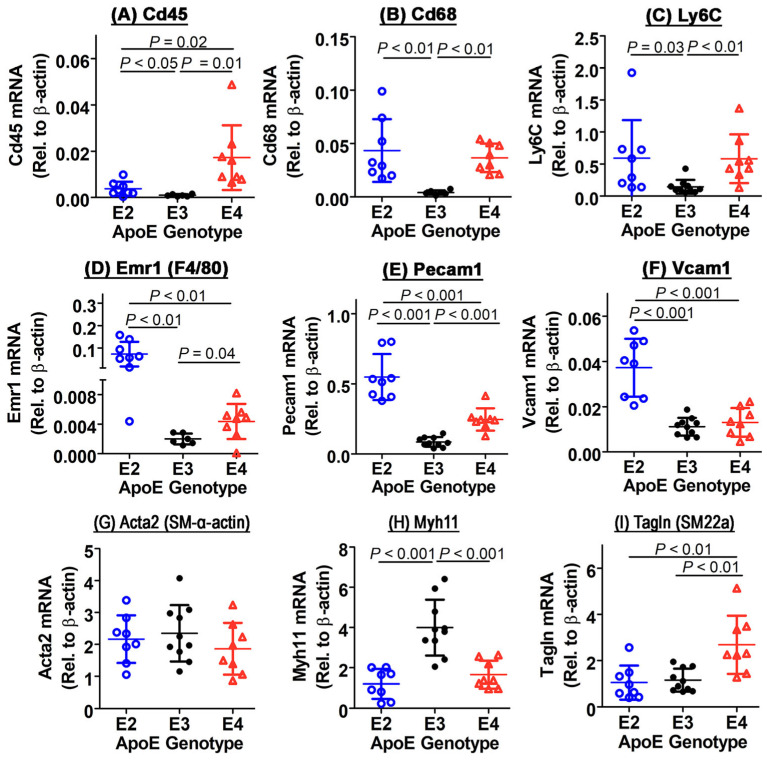
ApoE isoform-specific effects on vascular gene expression. Total RNA was prepared from the whole aorta of Western diet-fed *APOE2* (*n* = 8), *APOE3* (*n* = 6), and *APOE4* (*n* = 8) mice for quantitative RT-PCR to analyze expression of (**A**) *Cd45*, (**B**) *Cd68*, (**C**) *Ly6c*, (**D**) *Emr1*, (**E**) *Pecam1*, (**F**) *Vcam1*, (**G**) *Acta2*, (**H**) *Myh11*, and (**I**) *Tgln*. The data were evaluated by one-way ANOVA with Student–Newman–Keuls post hoc analysis and are presented as mean ± SD. Groups that are statistically different are shown with *p* values as indicated.

In addition to differences in plasma lipid levels that may influence atherosclerosis susceptibility, we have also reported previously that Western diet-fed *APOE2* mice displayed elevated levels of leukocytes, including monocytes and neutrophils but not lymphocytes, in blood compared to *APOE3* and *APOE4* mice [25]. Thus, the increased atherosclerosis observed in *APOE2* mice may be due to both hyperlipidemia and increased inflammation in these animals. The impact of hyperlipidemia and inflammation on the vasculature was explored initially by examining gene expression in the whole aortas of Western diet-fed *APOE2*, *APOE3*, and *APOE4* mice. Results showed that in comparison to *APOE3* mice, the aortas in *APOE2* mice also displayed elevated levels of *Cd45* expression, suggesting that the higher levels of circulating leukocytes in *APOE2* mice led to their infiltration into the vessel wall (Figure 2A). Interestingly, despite the similarity in leukocyte blood counts between Western diet-fed *APOE3* and *APOE4* mice [25], the aortas of *APOE4* mice also showed higher expression levels of *Cd45* compared to both *APOE2* and *APOE3* mice (Figure 2A), suggesting that the aortas of Western diet-fed *APOE4* mice were also inflamed. Additional analysis revealed increased expression of *Cd68*, *Ly6c*, and *Emr1* (also known as F4/80) genes indicative of elevated presence of inflammatory monocytes and macrophages in the aortas of *APOE2* and *APOE4* mice compared to *APOE3* mice (Figure 2B–D). The increase in leukocyte infiltration into the vessel wall was likely due to higher expression levels of *Pecam1* and *Vcam1*, two endothelial cell surface proteins that mediate monocyte adhesion and migration [32], in the aortas of *APOE2* mice as well as elevated expression of *Pecam1* in *APOE4* mice (Figure 2E,F). These data indicated that the aortas of both Western diet-fed *APOE2* and *APOE4* mice were highly inflamed in comparison to *APOE3* mouse aortas. In addition to differences in expression of inflammatory genes in the aortas of Western diet-fed *APOE2*, *APOE3*, and *APOE4* mice, expression of smooth muscle-specific genes was also found to be different between these animals. In particular, whereas expression levels of the smooth muscle cytoskeleton protein gene *Acta2* (also known as smooth muscle α-actin) were comparable among all three groups of mice, expression levels of the smooth muscle contractile protein myosin heavy chain 11 (*Myh11*) were reduced in *APOE2* and *APOE4* mice (Figure 2G,H), coinciding with their increase in leukocyte adhesion gene expression. In contrast, expression levels of another contractile protein, transgelin (*Tgln*, also known as SM22α), were found to be elevated in *APOE4* mice compared to *APOE2* and *APOE3* mice (Figure 2I). Taken together, we interpret these data to indicate apoE isoform-specific influence on vascular inflammation as well as the modulation of vascular cell phenotype in response to Western diet feeding.

### 2.3. ApoE Isoform-Dependent Differences in Cell Composition in the Aorta of Western Diet-Fed Mice

**Figure 3:**
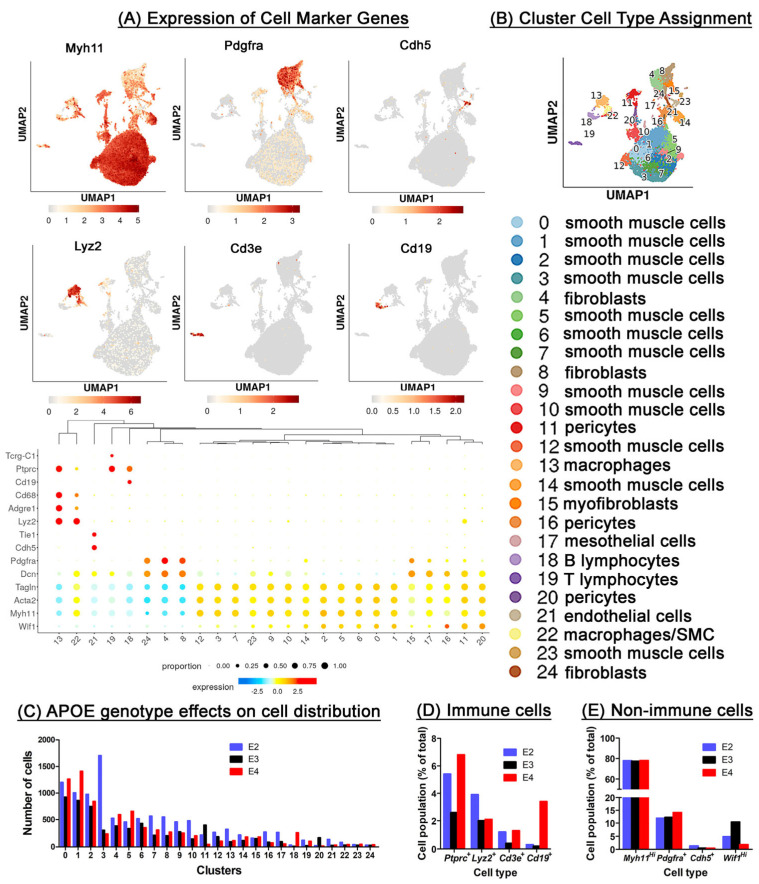
ApoE isoform-specific effects on vascular cell composition. Single cells were isolated from the whole aortas of Western diet-fed *APOE2*, *APOE3*, and *APOE4* mice. Cells from each genotype were pooled and subjected to single-cell RNA-seq analysis. (**A**) Uniform Manifold Approximation and Projection (UMAP) plots of cell-specific marker genes, revealing 25 different cell types and subsets identified in (**B**). (**C**) Bar graph showing the number of cells in each cell population of *APOE2*, *APOE3*, and *APOE4* mice. (**D**) Bar graph showing the percentage of all leukocytes (*Ptprc^+^*), including *Lyz2^+^* macrophages, *Cd3e^+^* T lymphocytes, and *Cd19^+^* lymphocytes in the aortas of APOE2, APOE3, and APOE4 mice. (**E**) Bar graph showing the percentage of smooth muscle cells (*Myh11^Hi^*), fibroblasts (*Pdgfa^+^*), endothelial cells (*Cdh5^+^*), and pericytes (*Wif1^Hi^*) in the aortas of *APOE2*, *APOE3*, and *APOE4* mice.

The influence of the various apoE isoforms on vascular cell composition and atherosclerosis development was examined in more detail by generating single-cell RNA-seq data from the aortas of male *APOE2*, *APOE3*, and *APOE4* mice after feeding them the Western diet for 16 weeks. A total of 10,500 cells from *APOE2* mice, 6264 cells from *APOE3* mice, and 7680 cells from APOE4 mice were analyzed. Unbiased cluster analysis revealed 25 clusters of cells. The cell clusters were further classified based on expression of canonical cell type-specific genes, such as *Ptprc/Cd45* as marker for immune cells, including the *Lyz2*-expressing macrophages and macrophage-like cells, *Cd3e*-expressing T lymphocytes, and *Cd19*-expressing B lymphocytes, as well as *Myh11* as a marker for smooth muscle cells, *Pdgfra* for fibroblasts, *Cdh5* for endothelial cells, and *Wif1^hi^* for pericytes or pericyte-like cells (Figure 3A). These cluster analyses identified one B lymphocyte cluster, a T lymphocyte cluster, and two clusters of cells expressing *Lyz2*, *Cd68*, and *Adgre1/Emr1* (F4/80) genes that are typical in macrophages. One of these macrophage clusters, cluster 22, also expressed smooth muscle cell marker genes such as *Acta2*, *Tagln*, and *Myh11*, which are likely smooth muscle cell-derived macrophage-like cells. In addition to these immune cell clusters, we also identified one endothelial cell cluster based on expression of *Cdh5* and *Tie1* genes, three clusters of cells that can be identified as fibroblasts with high expression of *Pdgfra* and *Dcn*, as well as three clusters of pericytes or pericyte-like cells with high expression of *Wif1*. All other gene clusters displayed high expression levels of *Acta2*, *Tagln*, and *Myh11* and were thus classified as various smooth muscle subsets or transitional smooth muscle cells from fibroblasts, pericytes, or endothelial cells (Figure 3B).

The proportions of cells within each cluster differed according to the *APOE* genotype. Consistent with the global gene expression data that showed increased *Cd45* expression in the aortas of *APOE2* and *APOE4* mice compared to *APOE3* mice, the scRNA-seq data also revealed more immune cells in the aortas of both *APOE2* and *APOE4* mice compared to *APOE3* mice (Figure 3C). Specifically, both APOE2 and *APOE4* mice displayed an increased number of *Cd3e^+^* T lymphocytes in their aortas compared to *APOE3* mice. However, more *Lyz2^+^* macrophages were present in the *APOE2* mouse aorta, whereas the *APOE4* mouse aorta displayed more *Cd19^+^* B lymphocytes (Figure 3D). Within the non-immune vascular cell population, a higher percentage of Wif1^Hi^ pericytes was present in *APOE3* mouse aorta, but the *APOE2* mice contained a higher percentage of *Cdh5^+^* endothelial cells compared to *APOE3* and *APOE4* mice (Figure 3E).

### 2.4. Macrophage Repertoire Differences in the Aortas of Western Diet-Fed APOE Gene Replacement Mice

**Figure 4:**
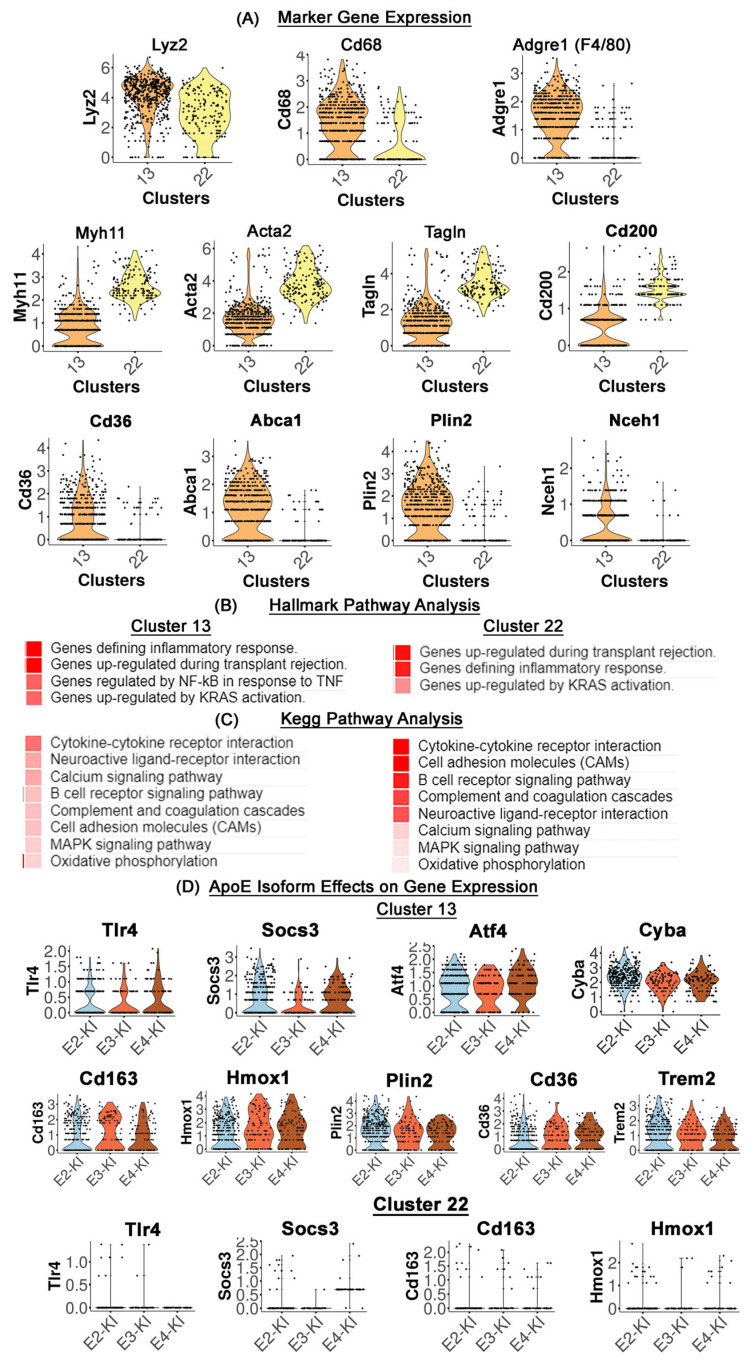
ApoE isoform-specific effects on macrophage subsets in aorta. (**A**) Violin plots with data points showing marker genes used to annotate various macrophage subtypes. (**B**) Hallmark Pathway analysis and (**C**) Kegg Pathway analysis of cluster 13 and cluster 22 macrophage subsets. The intensity of the color gradient depicts prevalence of activated genes in each pathway. (**D**) ApoE isoform effects on expression of specific genes in cluster 13 and cluster 22 cells in the aortas of Western diet-fed *APOE2*, *APOE3*, and *APOE4* mice.

Two clusters of cells, clusters 13 and 22, displaying high *Lyz2* and *Cd68* expression, were classified as macrophages or macrophage-like cells (Figure 3A). Cells in cluster 13 also showed higher expression of *Adgre1* (also known as *EMR1* or F4/80) but relatively low expression of smooth muscle cell-specific genes *Myh11*, *Acta2*, and *Tagln* (Figure 3A and Figure 4A). Therefore, cells in this cluster were likely classical macrophages derived from monocytes infiltrated into the aortas in response to Western diet feeding. In contrast, cells in cluster 22 showed very low *Adgre1* expression but displayed high expression levels of smooth muscle marker genes such as *Acta2* and *Tagln* (Figure 3A and Figure 4A). These characteristics are similar to those described for smooth muscle-derived macrophage-like foam cells in atherosclerotic lesions [33]. The identification of cluster 22 cells as smooth muscle-derived macrophage-like foam cells was further supported by expression of *Cd200* (Figure 4A), a cell surface lineage marker of vascular smooth muscle cells [34], as well as their relatively low expression of genes responsible for lipid and cholesterol droplet turnover such as *Cd36*, *Abca1*, *Plin2*, and *Nceh1* compared to macrophages in cluster 13 (Figure 4A). Therefore, cells in cluster 22 were likely smooth muscle-derived macrophage-like foam cells in response to the Western diet. Hallmark and Kegg Pathway analyses revealed high expression of inflammatory genes in cells within both clusters, including the enrichment of genes responsible for KRAS activation, cytokine–cytokine receptor interaction, cell adhesion molecules, complement and coagulation activation cascades, and mitogen-activated protein (MAP) signaling (Figure 4B,C). Both cluster 13 and cluster 22 cells were also enriched with genes responsible for oxidative phosphorylation (Figure 4B,C), suggesting the presence of alternatively activated or anti-inflammatory macrophages. However, the enrichment of oxidative phosphorylation genes may also reflect high lipid handling activity of pro-inflammatory macrophages in response to Western diet feeding [35,36].

The scRNA-seq data from clusters 13 and 22 macrophage-like cells were further analyzed based on APOE genotype of the samples. Consistent with pathway analysis of the data revealing enrichment of inflammatory response genes in cluster 13 cells, the pro-inflammatory genes such as *Tlr4* and *Socs3* were highly expressed in cluster 13 cells from Western diet-fed *APOE2*, *APOE3*, and *APOE4* mice (Figure 4D). However, these genes were expressed at higher levels in *APOE2* and *APOE4* mice compared to that in *APOE3* mice (Figure 4D). Expression levels of the endoplasmic reticulum-stress marker *Atf4* and the oxidative stress-marker *Cyba* were also higher in *APOE2* and *APOE4* cells compared to *APOE3* cells, whereas expression levels of anti-inflammatory and anti-oxidative stress-related *Cd163* and *Hmox1* were higher in cluster 13 cells from *APOE3* mice compared to *APOE2* and *APOE4* mice (Figure 4D). Although cluster 13 cells in both *APOE2* and *APOE4* mice exhibited characteristics of endoplasmic and oxidative stress, expression levels of the lipid metabolism genes such as *Cd36* and *Plin2* were higher in cluster 13 cells from *APOE2* mice but lower in *APOE4* mice compared to *APOE3* mice (Figure 4D). The difference in *Plin2* expression between *APOE2* and *APOE4* mice suggested that the endoplasmic- and oxidative stress in cluster 13 cells of *APOE2* mice was most likely due to increased lipid accumulation, whereas the stress in *APOE4* cluster 13 cells was independent of lipid storage but a direct effect of apoE4 on endoplasmic reticulum and oxidative stress [26]. This conclusion was further supported by the observation of high expression of the foam cell-promoting lipid receptor *Trem2* [37] in *APOE2* but not *APOE3* or *APOE4* cluster 13 cells (Figure 4D). It is of note that high *Trem2* expression is generally associated with anti-inflammatory response and oxidative phosphorylation [38]. Thus, the elevated oxidative phosphorylation pathway identified in cluster 13 cells may be related to *Trem2*-expressing cells. However, increased lipid-induced *Trem2* expression has also been shown to activate macrophage signaling pathways that enhance foam cell formation, pro-inflammatory cytokine production, and atherosclerosis progression [39,40]. Thus, the abundance of cluster 13 cells in *APOE2* mice may be responsible at least in part to the atherosclerosis observed in these animals.

In contrast to cluster 13 cells, *Tlr4*, *Socs3*, *Cd163*, and *Hmox1* levels were minimally detected in cluster 22 macrophage-like cells regardless of the *APOE* genotype (Figure 4D). The nearly absent expression of these classical macrophage inflammatory marker genes further distinguished cluster 22 cells from classical macrophages, which supported their identification as *Cd200*-expressing smooth muscle cell-derived macrophage-like foam cells. Analysis of the data revealed a higher number of cluster 22 cells in the aortas of *APOE2* mice compared to those from *APOE3* and *APOE4* mice, thus indicating increased proliferation and/or differentiation of smooth muscle cells to foamy macrophage-like cells after lipid accumulation (Figure 3C). Taken together, these results indicate that the *APOE* genotype has distinct influence on aortic macrophage inflammation, with *APOE2* and *APOE4* increasing polarization of the classical macrophages toward the M1 pro-inflammatory phenotype while reducing M2 anti-inflammatory polarization compared to *APOE3*. Moreover, these analyses also revealed that inflammation in macrophage-like cells derived from smooth muscle cells proceeded via mechanism independent of classical M1 and M2 polarization pathways.

### 2.5. Lymphocyte Subset Differences in Aortas of Western Diet-Fed APOE Gene Replacement Mice

**Figure 5:**
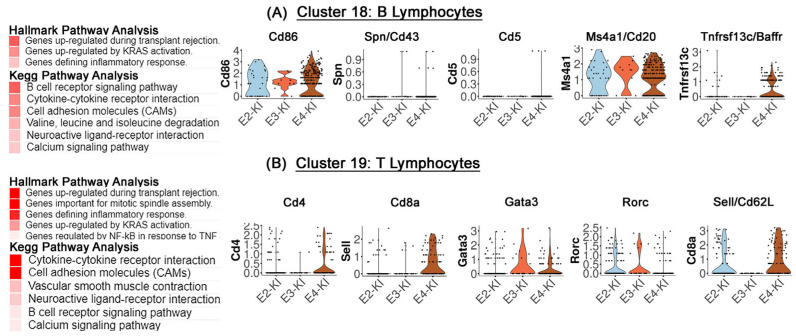
ApoE isoform-specific effects on lymphocyte composition in the aorta. Violin plots with data points showing apoE isoform-specific effects on expression of marker genes used to identify cluster 18 cells as B lymphocytes (**A**) and cluster 19 as T lymphocytes (**B**) in the aortas of *APOE2*, *APOE3*, and *APOE4* mice. High expression genes in each cluster were profiled and analyzed by Hallmark and Kegg Pathway analyses.

Cells in cluster 18 were identified as B lymphocytes based on *Cd19* expression (Figure 2A). While *Cd19^+^* cells represent only a small population of cells in the aortas of these Western diet-fed mice, the number of cluster 18 cells in *APOE4* mice exceeded those in *APOE2* and *APOE3* mice by ~10-fold (Figure 3C). Hallmark and Kegg Pathway analyses revealed enrichment of inflammatory response genes in cluster 18 cells from these Western diet-fed mice (Figure 5A). Additional data mining revealed high level *Cd86* expression in *APOE2* and *APOE4* mice compared to *APOE3* mice, suggesting the elevated presence of activated B lymphocytes in the aortas of both *APOE2* and *APOE4* mice (Figure 5A). Cells in this cluster displayed high expression levels of *Ms4a1/Cd20*, a marker of B-2 lymphocytes, but expression of the B-1 lymphocyte markers *Spn*/*Cd43* and *Cd5* were minimally detected in all cluster 18 cells (Figure 5A). Thus, most of the B lymphocytes in the aorta were the pro-atherogenic B-2 lymphocyte subtype after Western diet feeding [41]. The level of *Ms4a1/Cd20* expression was more prevalent in cluster 18 cells from *APOE2* and *APOE4* mice compared to *APOE3* mice (Figure 5A), suggesting that apoE2 and apoE4 may also promote atherogenesis via increased B-2 cells in the aorta. Unexpectedly, expression of *Tnfrs13c/Baffr* gene, encoding the B cell-activating factor receptor BAFFR, was found to be higher only in *APOE4* mice but not in *APOE2* mice (Figure 5A). Although the significance of the *APOE* genotype-dependent difference in BAFFR expression in B-2 lymphocytes remains unclear, these findings suggest that apoE2 and apoE4 expression may promote B-2 lymphocyte accumulation in aortas via different mechanisms.

In contrast to cluster 18 B lymphocytes, cluster 19 cells were identified as T lymphocytes based on high *Cd3e* gene expression (Figure 3). These cells were likely infiltrated into the aortas in response to inflammation after Western diet feeding. Indeed, Hallmark and Kegg Pathway analyses revealed high expression of inflammatory response genes in cluster 19 cells (Figure 5B). However, cluster 19 cells accounted for only a small percentage of cells in the aortas, which reflects the minimal atherosclerotic lesions detected in these animals under the experimental conditions of the current study. Nevertheless, detailed analysis of gene markers for T cell subsets revealed important differences in the T lymphocyte composition in the aortas of Western diet-fed *APOE2*, *APOE3*, and *APOE4* mice. In particular, *Cd4^+^* T helper cells were present in the aortas of *APOE4* mice but were minimally present in *APOE2* and *APOE3* mice. In contrast, *Cd8^+^* cytotoxic T cells were present in both *APOE2* and *APOE4* mice (Figure 5B). The near absence of *Cd4^+^* and *Cd8^+^* lymphocytes in the aortas of *APOE3* mice were consistent with 5-fold less cluster 19 cells and the resistance to vascular inflammation and atherosclerosis in these animals. Differences in atherosclerosis and inflammation in the aortas of these animals were likely not related to differences in inflammation suppression as the regulatory T cell transcription factor *FoxP3* was not detected in cluster 19 cells regardless of ApoE genotype. However, despite the detection of a low number of *Cd4^+^* T helper cells, the Th2-defining transcription factor *Gata3* [42] was found to be expressed at higher levels in cluster 19 cells from *APOE3* and *APOE4* mice compared to *APOE2* mice (Figure 5B). In contrast, the Th17-defining transcription factor *Rorc* [43] was higher in cells from *APOE2* and *APOE3* mice with minimal expression in *APOE4* mice. Likewise, the memory T cell marker Sell encoding *Cd62L* (also known as L-selectin) was detected only in cluster 19 cells from *APOE4* mice and was minimally expressed in cells from *APOE2* and *APOE3* mice (Figure 5B). Since apoE is not expressed in lymphocytes [44,45], differences in apoE isoform signaling in the vessel wall are likely responsible for the different lymphocyte subset composition in the aortas of Western diet-fed *APOE2*, *APOE3*, and *APOE4* mice. The differences in lymphocyte subset composition in the aortas may at least in part contribute to differences in inflammation and atherosclerosis susceptibility between *APOE2*, *APOE3*, and *APOE4* mice.

### 2.6. Endothelial Cell Differences in the Aortas of APOE2, APOE3, and APOE4 Mice

**Figure 6:**
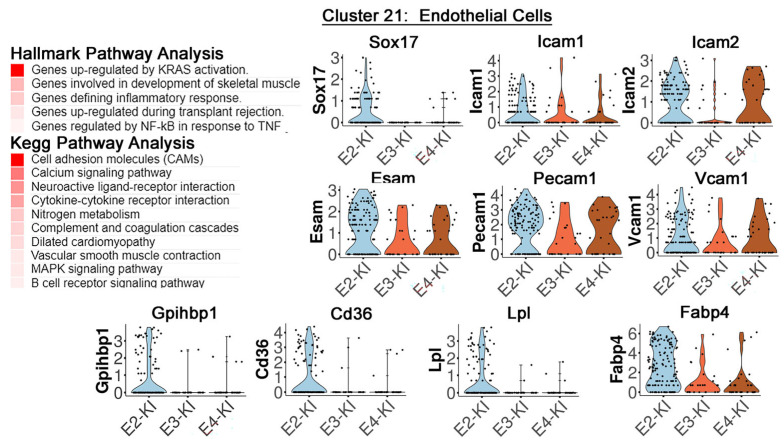
ApoE isoform-specific effects on aortic endothelial cells. Single-cell RNA-seq analysis revealed cluster 21 as endothelial cells. Hallmark and Kegg Pathway analyses revealed high expression of cell activation and inflammatory genes. The endothelial cell activation genes (*Sox17*, *Icam1*, *Icam2*, *Esam*, *Pecam1*, and *Vcam1*) and intracellular lipid uptake and processing genes (*Gpihbp1*, *Cd36*, *Lpl*, and *Fabp4*) were expressed in an apoE isoform-dependent manner.

Cells in cluster 21 were identified as endothelial cells based on *Cdh5* and *Tie1* gene expression (Figure 3A). While cell recovery was relatively low, approximately 5-fold more cluster 21 cells were recovered from aortas of *APOE2* mice compared to *APOE3* mice and 4-fold more than those obtained from *APOE4* mice. The higher number of cluster 21 endothelial cells in the aortas of *APOE2* mice was consistent with the higher expression levels of *Sox17* (Figure 6), a protein that plays an important role in endothelial adaptation to hemodynamic changes in response to fluid shear stress as well as vascular remodeling and vasculogenesis [46,47]. Hallmark and Kegg Pathway analyses revealed enrichment of inflammation genes that reflect vascular inflammation in response to Western diet feeding (Figure 6). In particular, high expression levels of *Icam1*, *Icam2*, and *Esam* which are responsible for leukocyte adhesion [48,49], *Pecam1* which facilitates leukocyte transmigration through the vessel wall [50], and *Vcam1* which promotes monocyte accumulation [51] were detected in cluster 21 endothelial cells from the aortas of *APOE2*, *APOE3*, and *APOE4* mice (Figure 6). Importantly, higher expression of these adhesion molecules was observed in endothelial cells from *APOE2* mice compared to *APOE3* and *APOE4* mice (Figure 6). The increased inflammatory gene expression in the endothelial cells of *APOE2* mice may be due to the high expression levels of lipid uptake genes such as *GPIHBP1*, *CD36*, and *LPL* as well as the intracellular fatty acid transporter gene *Fabp4* (Figure 6). The increased lipid uptake as well as expression of adhesion molecules in *APOE2* mouse endothelial cells may be a contributing factor to the higher percentage of macrophages and increased atherosclerotic lesions observed in *APOE2* mice.

### 2.7. APOE Genotype Differentially Affects Fibroblast and Fibroblast-like Cell Composition in Mouse Aorta

**Figure 7:**
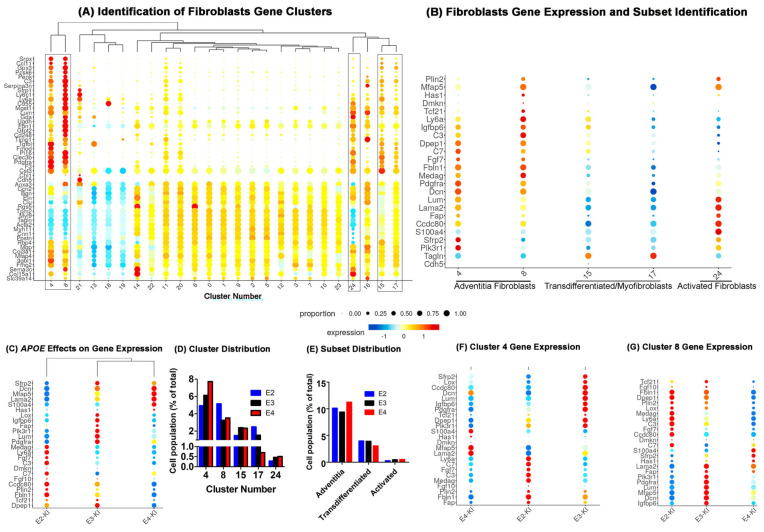
ApoE isoform-specific influence on aortic fibroblasts subsets and gene expression. (**A**) Expression of gene markers for fibroblasts, fibroblast-like cells, and smooth muscle cells in all 25 aortic cell clusters. The clusters identified as fibroblasts are boxed. (**B**) Expression of fibroblasts genes in the five fibroblasts subsets. (**C**) Expression of fibroblasts and fibroblast-related genes in the aortas of Western diet-fed *APOE2*, *APOE3*, and *APOE4* mice. (**D**) Bar graph showing the percentage of total aortic cells in fibroblasts and fibroblast-like cell clusters in *APOE2*, *APOE3*, and *APOE4* mice. (**E**) Bar graph showing the percentage of various fibroblasts subsets in *APOE2*, *APOE3*, and *APOE4* mice. (**F**) Expression levels of selected fibroblast gene markers in cluster 4 cells from *APOE2*, *APOE3*, and *APOE4* mice. (**G**) Expression levels of selected fibroblast gene markers in cluster 8 cells from *APOE2*, *APOE3*, and *APOE4* mice.

Five clusters of cells, namely clusters 4, 8, 15, 17, and 24, displayed relatively high *Pdgfra* and *Dcn* expression levels compared to cells in other clusters, suggesting that cells in these clusters may be classified as fibroblasts or fibroblast-like cells (Figure 7A). Additional analysis focusing on the expression of fibroblast gene markers, published previously [52,53,54,55], identified cells in clusters 4 and 8 which were closely related with high expression of fibroblast gene markers such as *Pdgfra*, *Dcn*, and *Ly6a* with low or minimal expression of smooth muscle-specific genes *Tagln*, *Acta2*, and *Myh11* or the endothelial-specific gene *Cdh5* (Figure 7B). Therefore, cells in clusters 4 and 8 were identified as subsets of adventitia fibroblasts. In contrast, cells in clusters 15 and 17 were related but distinct from the classical fibroblasts due to their high expression levels of gene markers for both fibroblasts and smooth muscle cells. Accordingly, cells in clusters 15 and 17 likely originated as smooth muscle cells and obtained fibroblastic characteristics as a result of transdifferentiation [56]. Alternatively, these cells may also be myofibroblasts that gained smooth muscle cell characteristics with Western diet feeding [52,53,55]. Finally, cluster 24 cells also expressed fibroblast gene markers with minimal expression of smooth muscle genes (Figure 7B). However, cluster 24 cells expressed higher levels of S100A4 and FAP with relatively low level expression of *Dcn*. Thus, cells in cluster 24 were probably activated fibroblasts in transition to myofibroblasts (Figure 7B). Unfortunately, cluster 24 cells cannot be definitively characterized due to their low cell number (<1% of the total cell population) in the aortas.

It is interesting to note that *APOE* genotype also influences the expression of genes responsible for fibroblast activation. In comparison to fibroblasts and fibroblast-like cells in the aorta of *APOE3* mice, which expressed high levels of *Lum*, *Igfbp6*, and *Fap* genes, fibroblastic cells in *APOE4* mice displayed high expression levels of *Mfap5*, *Lama2*, *Dcn*, and *S100a4* (Figure 7C). The fibroblasts and fibroblast-like cells in *APOE2* mouse aorta were different from cells in *APOE3* and *APOE4* mice and displayed high expression levels of genes that are expressed in lipofibroblasts such as *Plin2*, *Ly6a*, *Medag*, *Fbln1*, *Fgf7*, and *C3* (Figure 7C). Additionally, *APOE* genotype also influences cell distribution among the various fibroblast and fibroblast-like subsets. Despite similar percentage of fibroblasts and fibroblast-like cells in the aortas of Western diet-fed *APOE2*, *APOE3*, and *APOE4* mice, the aortas of *APOE4* mice contained a higher percentage, whereas the aortas of *APOE2* mice displayed a lower percentage of cluster 4 adventitia fibroblasts compared to the aortas of *APOE3* mice (Figure 7D,E). In contrast, a higher percentage of clusters 8 adventitia fibroblasts and cluster 17 transitional fibroblasts, but lower percentage of clusters 4 and 15 cells, were observed in *APOE2* mice compared to *APOE3* and *APOE4* mice (Figure 7D). Taken together, both *APOE2* and *APOE4* mice contained a higher percentage of adventitia fibroblasts compared to *APOE3* mice, and less transdifferentiated fibroblasts were observed in the aortas of *APOE4* mice (Figure 7E).

*APOE* genotype also appeared to affect gene expression profile within each cluster, particularly in the typical fibroblasts within clusters 4 and 8. Specifically, cluster 4 cells from *APOE2* mouse aorta were more related to those from *APOE3* mouse aorta, except for the high expression levels of genes that participate in intracellular lipid accumulation, whereas cells from *APOE4* mice were more distantly related due to preponderance of cells with high *S100a4*, *Mfap5*, and *Lama2* expression levels (Figure 7F). In contrast, whereas genes related to intracellular lipid accumulation were also highly expressed and prevalent in cluster 8 cells from *APOE2* mice, cluster 8 cells from *APOE3* mice were more similar to those from *APOE4* mice compared to those from *APOE2* mice (Figure 7G). For example, cluster 8 cells in both *APOE3* and *APOE4* mice displayed high expression levels of the fibroblasts activating factor gene *Fap*, whereas *Fap* was expressed at low levels in cluster 8 cells from *APOE2* mice. Interestingly, elevated expression of the pro-inflammatory and pro-fibrotic gene *S100a4* was only observed in cluster 8 cells from *APOE4* mice but not in *APOE3* and *APOE4* mice. We interpret these observations to indicate that cluster 8 cells in *APOE3* and *APOE4* mice were both activated fibroblasts, but their activation appeared to proceed via distinct mechanisms.

### 2.8. APOE Genotype Influence on Aortic Smooth Muscle Cell Phenotype Composition

**Figure 8:**
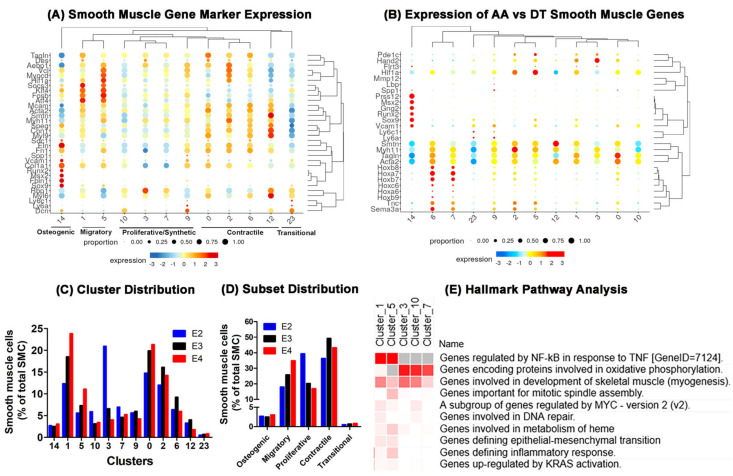
ApoE isoform-specific effects on aortic smooth muscle cells. (**A**) Expression profile of smooth muscle cell gene markers in 12 different smooth muscle cell subsets. (**B**) Expression profile of gene markers for aortic arch (AA) and descending thoracic aorta (DT) smooth muscle cells. (**C**) apoE isoform-specific effects on smooth muscle cell distribution among the 12 smooth muscle cell subsets. (**D**) ApoE isoform-specific effects on smooth muscle cell subset composition. (**E**) Hallmark Pathway analysis of gene expression in the five cell clusters displaying apoE isoform-specific differences. Intensity of the color gradients indicates prevalence of activated genes in each pathway.

Fourteen cell clusters were initially identified as smooth muscle cells in the mouse aorta based on high expression of *Myh11*, *Tagln*, and *Acta2* genes with minimal expression of marker genes for lymphocytes, monocytes/macrophages, and fibroblasts (Figure 3A). However, cells in clusters 13 and 20 also displayed high Wif1 expression level and were therefore re-classified as pericytes. Of the remaining 12 cell clusters, the high expression levels of *Runx2*, *Msx2*, and *Sox9* identified cluster 14 as osteogenic smooth muscle cells [57], whereas high expression levels of *Hif1a* and *Klf4* in clusters 1 and 5 identified these cells as highly migratory smooth muscle cells [58]. The other smooth muscle cell clusters were identified as either proliferative/synthetic smooth muscle cells (clusters 10, 3, 7, and 9) or contractile smooth muscle cells (clusters 0, 2, 6, and 12) based on expression levels of the classical smooth muscle genes such as *Acta2*, *Smtn*, *Myh11*, *Speg*, *Cnn1*, *Myl9*, and *Smtn* (Figure 8A). Cells in cluster 12 also displayed high expression of *Rbp1*, indicative of a transitional smooth muscle cell type that appeared transiently with endothelial injury [59]. Cluster 23 was another unique subset of smooth muscle cells with high *Rbp1* expression level. However, cells in cluster 23 also displayed high *Dcn* expression levels but with low expression levels of the classical smooth muscle cells. Thus, cluster 23 cells were likely in a smooth muscle–myofibroblast transition state.

Additional analysis based on expression of genes localized to the aortic arch such as *Pde1c*, *Prss12*, *Gng2*, *Hand2*, *Flrt3*, *Lbp*, *Mmp12*, and *Spp1*, or genes expressed in the descending aorta such as the homeobox genes *Hoxb7*, *Hoxa7*, *Hoxb8*, *Hoxb9*, *Hoxa6*, *Hoxc6* as well as *Sema3a* and *Tnc* [60], revealed that cell clusters displaying similar phenotypes identified above were different subsets due to their anatomic location in the vascular bed. For example, the highly migratory smooth muscle cells in cluster 1 were derived mainly from the aortic arch, whereas similar cells in cluster 5 were a mixture of smooth muscle cells from the aortic arch and descending aorta (Figure 8B). Likewise, different cluster identification for the proliferative/synthetic smooth muscle cells was likely due to cell origin with cluster 3 cells derived from the aortic arch and clusters 7 and 9 cells derived from the descending aorta (Figure 8B). Similar analysis revealed that the contractile smooth muscle cell clusters were derived from mixed cell origin (clusters 0, 2) or were predominantly descending aorta smooth muscle cells (clusters 6, 12).

The aortas of APOE4 mice contained a higher percentage of highly migratory smooth muscle cells regardless of whether the cells were derived from the aortic arch or the descending aorta (clusters 1 and 5). In contrast, both the aortic arch and descending aortas of *APOE2* mice displayed a high percentage of proliferative/synthetic smooth muscle cells (clusters 10, 3, 7). The *APOE2* aortas also contained less cells in clusters 0 and 2 compared to aortas from *APOE3* and *APOE4* mice, whereas both *APOE2* and *APOE4* aortas contained lower percentage of cluster 6 smooth muscle cells compared to that found in *APOE3* aortas (Figure 8C). Since cells in clusters 0, 2, and 6 were identified as normal contractile smooth muscle cells, these results indicated that apoE2 and apoE4 expression increased de-differentiation of smooth muscle cells with reduced presence of normal contractile smooth muscle cells in the aorta (Figure 8D). Whereas apoE2 expression increased presence of proliferative/synthetic smooth muscle cells in the aorta, apoE4 expression elevated the presence migratory smooth muscle cells (Figure 8D). Consistent with this interpretation is that Hallmark Pathway analysis revealed increased expression of muscle development genes in clusters 1, 5, 3, 10, and 7 that were enriched in either *APOE2* or *APOE4* mice (Figure 8E). Hallmark Pathway analysis also revealed that cluster 1 and 5 cells enriched in *APOE4* mice displayed high expression levels of NFĸB-responsive genes as well as elevated expression of pro-oxidation and inflammatory genes (Figure 8E). In contrast, cluster 3, 10, and 7 cells enriched in *APOE2* mice were cells with high expression of genes in the oxidative phosphorylation pathway (Figure 8E). These include both mitochondrial-encoded genes as well as nuclear-encoded respiratory chain genes. A complete list showing changes of oxidative phosphorylation genes across all smooth muscle cell clusters is shown in Supplemental Materials (Table S1), while significant changes based on GSEA are shown in Supplemental Materials (Table S2). The summary of these results for the oxidative phosphorylation pathway across all smooth muscle cell clusters is shown in Table S3. Of particular interest was the enrichment of genes coding for NADH:ubiquinone oxidoreductase subunits that form mitochondria complex 1, such as *Ndufa6*, *Ndufb7*, *Ndufs1*, *Ndufs6*, *Ndufa9*, and *Ndufs3*, which were highly expressed in these clusters (Tables S1 and S2). The increased expression of these mitochondria complex 1 proteins may result in electron transport chain leakage and reactive oxygen species generation to promote smooth muscle cell dedifferentiation to a proliferative/synthetic phenotype [61,62]. Taken together, different cell clusters enriched in the aortas of *APOE2* and *APOE4* mice, each with their unique signaling pathway gene expression, suggested that apoE2 and apoE4 may promote vascular smooth muscle cell remodeling via distinct mechanisms.

## 3. Discussion

This study used gene replacement mouse models in which the endogenous mouse ApoE gene was replaced at the same locus with either human *APOE2, APOE3*, or *APOE4* genes and showed that human ApoE2 expression promotes hyperlipidemia and atherosclerosis in response to feeding mice a high fat–high cholesterol Western-type diet. These results are consistent with those observed in similar studies when the mice were fed an atherogenic diet containing high cholesterol and cholic acid [30], as well as human studies documenting the association between *APOE2* genotype with familial dysbetalipoproteinemia [63]. In contrast, Western diet-fed *APOE3* and *APOE4* mice did not display hyperlipidemia or atherosclerosis. These results were different from those reported earlier in which *APOE3* and *APOE4* mice were shown to be hyperlipidemic and developed atherosclerosis when fed the cholesterol-cholate diet [31,64]. The discrepancy between the current and earlier studies may be due to differences in the diet used. The diet used in our study mimics human diets, and the lack of atherosclerosis in *APOE3* mice was consistent with human studies, showing that in comparison with *APOE2* and *APOE4*, the *APOE3* genotype is associated with the lowest risk of cardiovascular disease. Interestingly, while atherosclerosis was not observed in the *APOE4* mice during the course of this 16-week diet study, *APOE4* genotype is associated with increased risk of cardiovascular disease in humans [7,8]. However, despite the lack of atherosclerosis in the *APOE4* mice, increased inflammation was observed in these animals, thus indicating that the *APOE4* mice were prone to developing atherosclerotic lesions with prolonged Western diet feeding. The *APOE2* mice also exhibited increased inflammation in addition to hyperlipidemia and atherosclerosis, thus indicating that the elevated inflammation augmented hyperlipidemia to accelerate atherosclerosis in these animals during the course of this study.

The mechanism underlying the increased inflammation in the aortas of *APOE2* mice is different from that observed in *APOE4* mice. Specifically, the increased inflammation in *APOE2* mice was due primarily to hyperlipidemia affecting immune and vascular cells in these animals after Western diet feeding. In particular, elevated presence of classical monocyte-derived macrophages (cluster 13 cells) in the *APOE2* mice is consistent with previous reports of hypercholesterolemia-induced monocytosis [65] and myelopoiesis in *APOE2* mice [25]. The aortas of Western diet-fed *APOE2* mice also exhibited higher numbers of smooth muscle cell-derived macrophage-like foam cells (cluster 22) and aortic fibroblasts with high expression levels of intracellular lipid accumulation genes (cluster 4). The lipid accumulation in these macrophage-like foam cells and lipofibroblasts resulted in a pro-inflammatory phenotype that caused a higher number of activated smooth muscle cells with proliferative/synthetic characteristics that promote atherosclerosis. Additionally, the aortas of Western diet-fed *APOE2* mice also displayed a higher number of inflamed endothelial cells with elevated expression levels of adhesion molecules that facilitate leukocyte transmigration. The increased levels of adhesion molecules coincided with high expression of lipid uptake genes in the endothelial cells, thereby supporting the concept that lipid accumulation in endothelial cells also promotes vascular inflammation to accelerate atherosclerosis [66]. The increased lipid uptake by endothelial cells in the vessel wall of *APOE2* mice is likely due to impaired liver clearance and plasma accumulation of triglyceride- and cholesterol-rich atherogenic lipoproteins. Interestingly, the aortas of Western diet-fed *APOE2* mice also showed the presence of activated B-2 lymphocytes that promote atherosclerosis. However, despite their presence and indication of inflammation, the number of these B-2 cells in *APOE2* mice was significantly less than that observed in *APOE4* mice. The lower number of these B-2 cells in *APOE2* mice is likely due to impaired lipid antigen presentation that requires avid apoE-LDL receptor interaction [67].

In contrast to the *APOE2* mice, the elevated inflammation observed in *APOE4* mice was not related to hyperlipidemia and aberrant lipid storage in immune and vascular cells. For example, Western diet-fed *APOE4* mice displayed a similar number of classical monocyte-derived macrophages (cluster 13) as *APOE3* mice, thus indicating that apoE4 expression did not promote monocytosis and myelopoiesis, a hallmark of hyperlipidemia-induced inflammatory response. Moreover, cluster 13 cells in *APOE4* mice also exhibited lower levels of *Plin2* expression compared to cells from *APOE2* and *APOE3* mice, thus further indicating that cluster 13 cells in *APOE4* mice were not lipid-loaded. These results are in striking contrast to the *APOE4* effects in microglia, where lipid accumulation and a lipogenic program were observed [68,69]. While the cell type-specific differences remained unresolved, *APOE4* has been shown to reduce lipoprotein uptake in microglia but not in bone marrow-derived macrophages [68,70]. Regardless, despite the lack of lipid loading or an increase in cell number, cluster 13 macrophages in *APOE4* mice also exhibited a pro-inflammatory phenotype with increased expression of endoplasmic reticulum- and oxidative-stress genes compared to that observed in cluster 13 cells from *APOE3* mice. This observation is consistent with previous reports that showed oxidative stress-induced inflammatory response in apoE4-expressing myeloid cells [25]. Oxidative stress-induced inflammatory response in *APOE4* mice was also evident by the minimal expression of *Rorc* gene with elevated *Cd62L* expression, and thus low levels of Th17 cells, in the T lymphocyte population [71]. These T lymphocyte characteristics were not observed in *APOE2* and *APOE3* mice. Interestingly, although the aortas in both Western diet-fed *APOE2* and *APOE4* mice exhibited an increased number of *Cd8^+^* T lymphocytes compared to that observed in *APOE3* mice, the increased number of *Cd8^+^* cells in *APOE2* mice cannot be attributed to oxidative stress but likely a result of hyperlipidemia [72]. Taken together, these results support the concept that apoE2 and apoE4 promote innate and adaptive immune responses via distinct mechanisms.

Analysis of fibroblasts and fibroblast-like cells in the aortas of Western diet-fed mice provided additional evidence that the various apoE isoforms activate these cells via distinct mechanisms. In particular, the data presented in Figure 7 reveals that *APOE2* activates adventitial fibroblasts and their transition to lipofibroblasts with increased inflammation via elevated intracellular lipid storage as a consequence of hyperlipidemia. In contrast, *APOE4* does not cause lipid accumulation in aortic fibroblasts but elevates fibroblasts inflammation that favors fibrosis and negative remodeling, which also influences smooth muscle cell inflammation and differentiation. In contrast, *APOE3* activates fibroblasts with increased *Fap* expression without the corresponding increase in *S100a4*, thus providing an activated myofibroblast phenotype that favors atheroprotection [73,74]. These differences by which each apoE isoform activates vascular fibroblasts underscore additional mechanisms of how specific apoE isoform may influence atherosclerosis in response to the Western diet.

The smooth muscle cell subsets in the aortas were also influenced by expression of specific apoE isoforms. Regardless of whether the smooth muscle cells were derived from the aortic arch, which were neural crest-derived, or the descending aorta, which originated from the somites formed from the paraxial mesoderm, the predominant smooth muscle cell subsets in Western diet-fed *APOE3* mice were the quiescent contractile phenotype (clusters 0, 2, 6) with a population of de-differentiated smooth muscle cells with enhanced migratory characteristics (cluster 1). In contrast, these smooth muscle cells were less prominent in *APOE2* mice and were replaced with cells in clusters 3, 7 and 10, displaying high expression levels of genes associated with a proliferative/synthetic phenotype. The smooth muscle cell composition in the aortas of *APOE4* mice differed from that in *APOE2* and *APOE3* mice with enrichment of clusters 1 and 5 cells, which highly expressed genes involved with DNA synthesis, oxidative stress (heme metabolism), and inflammatory response. The differences in smooth muscle cell repertoire in these animals further illustrated distinct mechanisms by which apoE2, apoE3, and apoE4 modulate vascular remodeling and sensitivity to atherosclerosis in response to Western diet feeding. Nevertheless, this study with scRNA-seq analysis did not shed light on whether the effects on smooth muscle cell subset composition were due to apoE expressed intrinsically in the smooth muscle cells or whether the effects were influenced by circulating apoE or indirectly through apoE isoform influence on cytokine/chemokine secretion by other cells in the vasculature. In this regard, earlier in vitro studies have shown that exogenous apoE2 was less effective than apoE3, and apoE4 was ineffective in inhibiting smooth muscle cell proliferation [75]. Thus, the differences in smooth muscle cell subsets in *APOE2, APOE3,* and *APOE4* mice may be due at least in part to extracellular apoE in the plasma circulation. Whether apoE isoforms expressed endogenously in smooth muscle cells also influence cell phenotype characteristics remains to be determined.

## 4. Materials and Methods

### 4.1. Animals and Diets

Gene replacement mice, in which the endogenous mouse ApoE gene was replaced with the human *APOE2*, *APOE3*, or *APOE4* gene at the same locus, were obtained from the Maeda Laboratory at the University of North Carolina, USA [31,64] and backcrossed to C57BL/6J background. The mice were maintained on a 12 h light/dark cycle in our institutional pathogen-free animal care facility with free access to normal chow diet (Purina PicoLab Rodent Irradiated Diet 5053; Cincinnati Lab Supply, Cincinnati, OH, USA) and water. Age-matched (12-week-old) male mice were fed a Western diet containing 42% fat and 0.2% cholesterol (TD88137; Teklad Custom Diets, Madison, WI, USA) for 16 weeks. Blood and tissue samples were collected at the end of the experimental period for plasma lipid measurements, atherosclerosis assessment, and single-cell RNA sequencing analysis. All animal experiments were conducted in compliance with the U.S. National Institutes of Health guidelines) and were reviewed and approved by the University of Cincinnati Institutional Animal Care and Use Committee of the University of Cincinnati, in accordance with National Institutes of Health guidelines.

### 4.2. Plasma Lipid Measurement

Blood samples were collected from the tail vein after an overnight fast into EDTA-containing tubes. Plasma was prepared by centrifugation at 1500× *g* for 10 min at 4 °C. Plasma cholesterol and triglyceride levels were quantified by Infinity colorimetric assay kits (TR22421 and TR13421, Thermo Fisher Scientific, Cincinnati, OH, USA).

### 4.3. Atherosclerotic Lesion Analysis

Atherosclerosis was assessed in the whole aorta by *en face* analysis and cryosections of the aortic roots [76]. Briefly, mice were anesthetized and then perfused with phosphate-buffered saline containing 10% formalin prior to harvesting the heart and the entire aorta to the ileac bifurcation. The whole aortas were opened longitudinally from the bifurcation of the subclavian and carotid arteries to the iliac bifurcation and then stained with Oil Red O for 30 min for *en face* analysis. For analysis of the aortic roots, the hearts were fixed in 4% paraformaldehyde and embedded in Scigen Tissue-PlusTM OCT compound (Thermo Fisher Scientific, Cincinnati, OH, USA). Cryosections of 5 μm thickness were performed, starting at the valve nubs and the appearance of the coronary artery branch throughout the aortic sinus until the valve separates at the base of the heart. The cryosections were mounted on microscope slides and then stained with Oil Red O and counterstained with hematoxylin. All images were taken using an Olympus BX61 microscope and were subsequently quantified using ImageJ software, version 1.52 (National Institutes of Health, Bethesda, MD, USA).

### 4.4. Aortic mRNA Expression Analysis

Gene expression in the aorta was analyzed by quantitative real-time PCR. RNA was isolated from the whole aorta using TRIzol reagent (Invitrogen, Carlsbad, CA, USA), followed by cDNA synthesis using the qScript cDNA Synthesis Kit (QuantaBio, Beverley, MA, USA). Quantitative real-time PCR was performed with a StepOnePlus Thermocycler using FastSYBR Green Master Mix (Applied Biosystems, Carlsbad, CA, USA). Sequence-specific primers are listed in Table 1. Expression levels of mRNA were normalized to β-actin using the ΔΔCT analysis method.

### 4.5. Single-Cell RNA Sequencing

Mice were anesthetized and then perfused with phosphate-buffered saline. The whole aorta from the aortic arch to the ileac bifurcation was isolated and trimmed of excess fat tissues surrounding the aorta. The entire aorta was minced into small pieces in phosphate-buffered saline containing 2% BSA and then incubated for 1 h at 37 °C with gentle shaking in the same buffer containing 450 U/mL collagen-1, 120 U/mL collagen XI, 60 U/mL DNase 1, 60 U/mL hyaluronidase, 1.5 mM CaCl2, and 1.25 mg/mL elastase. The resulting cell suspension was filtered through 70 μm filters and washed several times to obtain single-cell suspension. Quality control was assessed by live/dead cell staining with trypan blue staining. Cell suspensions obtained from six APOE2, five APOE3, and seven APOE4 mice after 16 weeks of Western diet feeding were pooled according to their genotype for single-cell RNA-seq analysis. Approximately 10,000 cells recovered from each pooled cell suspension were loaded onto a 10× Genomics Chromium instrument (Pleasanton, CA, USA) to generate single-cell gel beads in emulsion and preparation of a bar-coded cDNA library for single-cell RNA-sequencing. Quality of the cDNA library was confirmed using an Agilent Bioanalyzer (Agilent Technologies, Santa Clara, CA, USA). Full-length sequencing was performed on two paired-end 75 bp flow cells using NovaSeq S4 platform (Illumina, San Diego, CA, USA) with an overall sequencing depth of approximately 400 million read pairs per sample.

### 4.6. Single-Cell RNA Sequencing Data Analysis

The 10× genomic single cell transcriptome sequencing data were processed using the Cell Ranger version 841 Single Cell software available from 10× Genomics (Pleasanton, CA, USA). The raw data was de-multiplexed, aligned to the *Mus musculus* (mm10) reference genome, and quantified using the Cell Ranger processing pipelines [77]. Further quality control filtering, normalization, integration and clustering were performed using the Seurat package (version 5.4.0; https://www.rdocumentation.org/) [78]. Quality control metrics for the overall scRNA-seq analysis, including nFeature, nCount, and percent mitochondrial reads are shown in Supplemental Figure (Figure S1). Cells with low count depth, few detected genes and high fraction of mitochondrial gene counts were removed. The data was normalized using regularized negative binomial regression as implemented in the latest version (version 2) of sctransform [79], integrated using the Seurat integration protocol based on anchoring and canonical correlation [78,80], clustered using the Louvain–Jaccard graph community detection algorithm [81,82], and visualized in two dimensions by UMAP dimensionality reduction [83]. Cluster-specific gene expression analysis was performed by aggregating regularized counts for each cluster and then performing single-tailed Limma-voom analysis [84] for up-regulated genes in the cluster of interest as compared to all other clusters. The pathway analysis of genes up-regulated in each cluster was performed by Gene Set Enrichment Analysis (GSEA) [85] as implemented in the R package *fgsea* (version 4.6, https://bioconductor.posit.co/packages/3.23/bioc/html/fgsea.html (accessed on 1 June 2026)) [86]. Pathway libraries used in GSEA included Kyoto Encyclopedia of Genes and Genomes (KEGG) pathways [87,88] and hallmark gene sets in MSigDb [89]. Additional analyses and visualizations were performed using ShinyCell (https://gihub.com/the-ouyang-lab/ShinyCell2 (accessed on 1 June 2026)) [90].

### 4.7. Statistics

All data were expressed as mean ± SD. Statistical analysis was performed using GraphPad Prism version 5.0 software (San Diego, CA, USA). All data passed the Shapiro–Wilk test for normality and were analyzed by one-way ANOVA with Student–Newman–Keuls post hoc analysis for multiple group comparisons. Differences at *p* < 0.05 were considered statistically significant.

## 5. Conclusions

In summary, while apoE-deficient mice has been shown to display an altered cellular landscape of the vessel wall with elevation of immune cells and progenitor cell-like smooth muscle cells compared to wild-type mice [91], this study documented for the first time that apoE also modulates vascular cell landscape in an isoform-specific manner. In particular, the data revealed that in comparison to apoE3 expression, apoE2 expression exacerbates diet-induced inflammation of infiltrating leukocytes, vascular endothelium, fibroblasts, and smooth muscle cells via mechanisms related to intracellular lipid accumulation, whereas apoE4 expression promotes vascular inflammation primarily through elevated oxidative and endoplasmic reticulum stress. The significance of this study is that the *APOE* genotype should be considered in designing intervention strategies to reduce the progression of atherosclerosis in cardiovascular disease.

### Limitations of the Study

It is important to note that there are several limitations of this study. Firstly, the entire aorta was used to characterize cell composition in the vessel wall in response to diet-induced atherosclerosis. Since atherosclerotic lesions in rodent models were typically observed only in the aortic roots with fatty lesions present in the thoracic aorta, the cellular repertoire within the atherosclerotic lesion may be different. Moreover, human atherosclerotic lesions can be present in several different arteries including coronary arteries, cerebral arteries, and renal arteries; future studies comparing cell composition and different cellular subsets in human atherosclerosis in various vascular regions may be worthwhile. Secondly, only mRNA expression level was assessed in the whole aorta and in scRNA-seq experiments. Future studies examining protein expression levels are necessary to validate these results. Finally, only male *APOE* gene replacement mice were examined in this study. In view of a recent study showing that *APOE* genetic variation impacts hepatic metabolism and mitochondrial activities in a sex-specific manner [92], additional studies with female mice may dissect potential sex-specific differences in the apoE isoform effect on vascular cell composition in the vessel wall.

## Figures and Tables

**Table 1:** Primer sequences used for PCR analysis.

Name	Forward Primer	Reverse Primer
β-actin	ACGGCCAGGTCATCACTATTG	CAAGAAGGAAGGCTGGAAAAGA
Cd45	GAGCATTCCACGGGTATTCAG	ACACGGTTATAATCATAGGGAAGAATGT
Cd68	TTTCTCCAGCTGTTCACCTTGA	CCCGAAGTGTCCCTTGTCA
Ly6c	TGCTATGGAGTGCCAATTGAGA	GAGCAATGCAGAATCCATCAGA
Emr1 (F4/80)	TGTCTGACAATTGGGATCTGCCCT	ATACGTTCCGAGAGTGTTGTGGCA
Pecam1	TGCGGTGGTTGTCATTGG	TGTTTGGCCTTGGCTTTCC
Vcam1	ATCTCCCCTGAATACAAAACGATT	GCCCGTAGTGCTGCAAGTG
Acta2 (SM-α-actin)	TCCCTGGAGAAGAGCTACGAACT	AAGCGTTCGTTTCCAATGGT
Myh11	TTCGCCCAAGTGACTTTTTTAGT	GGGTGCAAATAAGTGTGATTGC
Tagln (SM22a)	CACAAACGACCAAGCCTTCTC	TCGGCTCATGCCGTAGGA

## Data Availability

The data supporting this study are available in the article and are available from the corresponding author upon request. All single-cell RNA sequencing data have been deposited in the Gene Expression Ominbus (GSE314969) and are publicly available.

## Supplementary Materials

The following supporting information can be downloaded at: https://www.mdpi.com/article/10.3390/ijms27125619/s1.

## Abbreviations

The following abbreviations are used in this manuscript:

Apo	Apolipoprotein
ACTA2	Smooth muscle α-actin
BAFFR	B cell-activating factor receptor
CD36	Cluster of differentiation 36
CD45	Cluster of differenetiation 45
ESAM	Endothelial cell-selective adhesion molecule
FABP4	Fatty acid binding protein 4
GPIHBP1	Glycosylphosphatidylinositol anchored HDL binding protein 1
ICAM1/2	Intercellular adhesion molecule ½
LDL	Low density lipoprotein
LPL	Lipoprotein lipase
MAP	Mitogen-activated protein
MYH11	Myosin heavy chain 11
PECAM1	Platelet endothelial cell adhesion molecule 1
scRNA-seq	Single cell RNA-sequencing
SOX17	SRY-box transcription factor
TAGLN	Transgelin/SM22a
UMAP	Uniform manifold approximation and projection
VCAM1	Vascular cell adhesion protein 1
