# Supplementary material for: Aortic Single-Cell Transcriptome Analysis Reveals ApoE-Isoform-Specific Influences on Vascular Disease
Source: Int J Mol Sci. 2026 Jun 22;27(12):5619. doi: 10.3390/ijms27125619 (PMC13299737; doi:10.3390/ijms27125619)
Supplement: Supplementary file 1 [file ijms-27-05619-s001.zip › ijms-4323115-supplementary/Supplemental materials/Supplemental Figure S1 - quality control metrics.pdf]

Supplemental Figure S-1

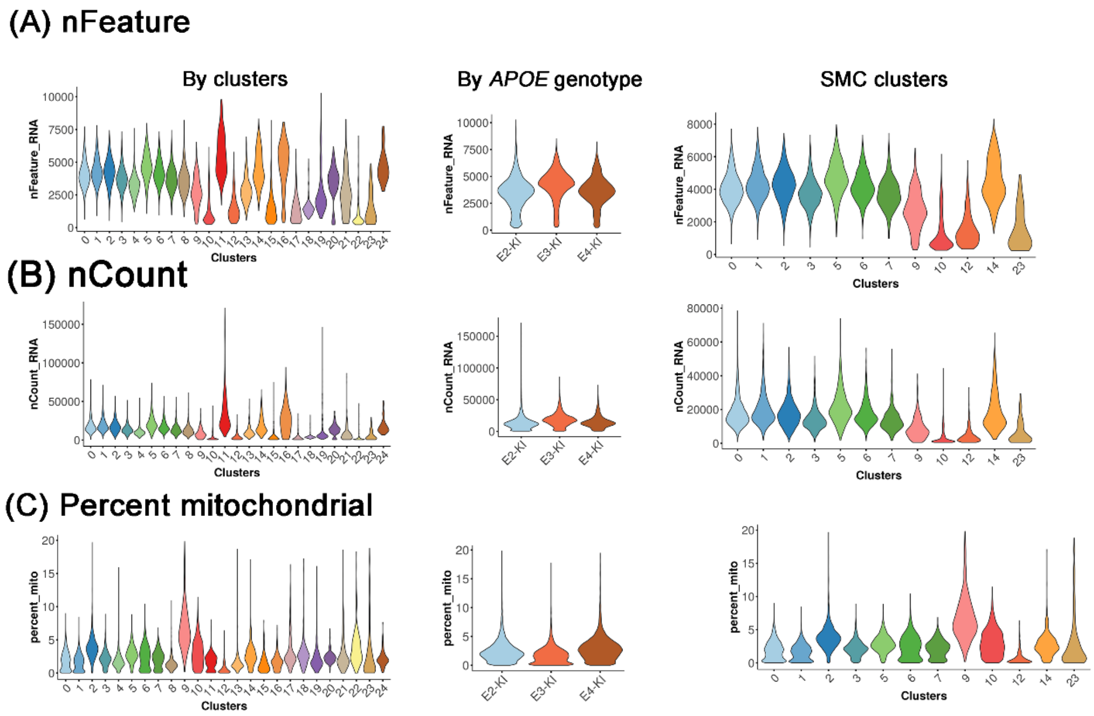

Figure S-1. Quality control metrics for scRNA-seq data analysis. The quality control metrics, including (A) nFeature, (B) nCount, and (C) percent mitochondrial reads are presented across individual cell clusters or *APOE* genotype, as well as across smooth muscle cell (SMC)-specific clusters.
